# First Case of *Ewingella americana* Meningitis in a Term Newborn: A *Rare* but *Real* Pathogen

**DOI:** 10.3389/fped.2020.00308

**Published:** 2020-06-12

**Authors:** Sarah Meisler, Ranjith Kamity, Asif Noor, Leonard Krilov, Caterina Tiozzo

**Affiliations:** ^1^Department of Pediatrics, NYU Winthrop Hospital, Mineola, NY, United States; ^2^Division of Neonatology, Department of Pediatrics, NYU Winthrop Hospital, Mineola, NY, United States; ^3^Department of Pediatrics, NYU Long Island College of Medicine, Mineola, NY, United States; ^4^Division of Infectious Diseases, Department of Pediatrics, NYU Winthrop Hospital, Mineola, NY, United States

**Keywords:** *Ewingella americana*, newborn, meningitis, emergent pathogens, neonatal sepsis, gram negative anaerobic bacteria

## Abstract

*Ewingella americana* is a Gram-negative, catalase positive and anaerobic enterobacterium first described in 1983. Infections caused by this pathogen, such as bacteremia and pneumonia, are extremely rare and primarily occur in patients with underlying pathologies or immunosuppression. There is still a debate as to whether *Ewingella americana* is a real pathogen or if it can be considered an opportunistic infectious agent. We report the first documented case of *Ewingella americana* meningitis in literature and the first case of this pathogen causing infection in a newborn.

**Case presentation:** A term newborn male was born via spontaneous vaginal delivery to a Gravida 2 Para 0, 28 year old woman with negative prenatal screening tests with a birth weight of 4.70 kilograms and Apgar scores of 9 and 9 at 1 and 5 minutes respectively. Rupture of membranes was 27 hours prior to delivery. Infant was noted to be febrile to 101°F at birth, so infant was admitted in the neonatal intensive care unit and started empirically on ampicillin and gentamycin. Cerebrospinal fluid (CSF) drawn due to irritability on day of life 1 presented normal cell and protein count but grew Gram negative rods after 2 days, identified subsequently as *Ewingella americana*; repeat CSF analysis done at 6 days of life showed pleocytosis. Brain MRI performed at 2 weeks of life showed leptomeningitis. The infant was treated with ceftazidime for 21 days from the first negative CSF culture. He has since followed up with the neurologist and infectious disease specialist. He had a normal electroencephalogram (EEG) and is meeting all developmental milestones at the 24 months of age follow up visit.

**Conclusion:** Our case highlights that *Ewingella americana* can cause serious invasive infections such as meningitis in the neonatal period with minimal symptomatology. Antibiotic treatment in the neonatal period can present challenges due to the *Ewingella americana*'s variable sensitivity. The role of these emerging low virulence organisms in causing infections has to be further elucidated, especially in vulnerable patients such as newborns.

## Introduction

**Figure 1:**
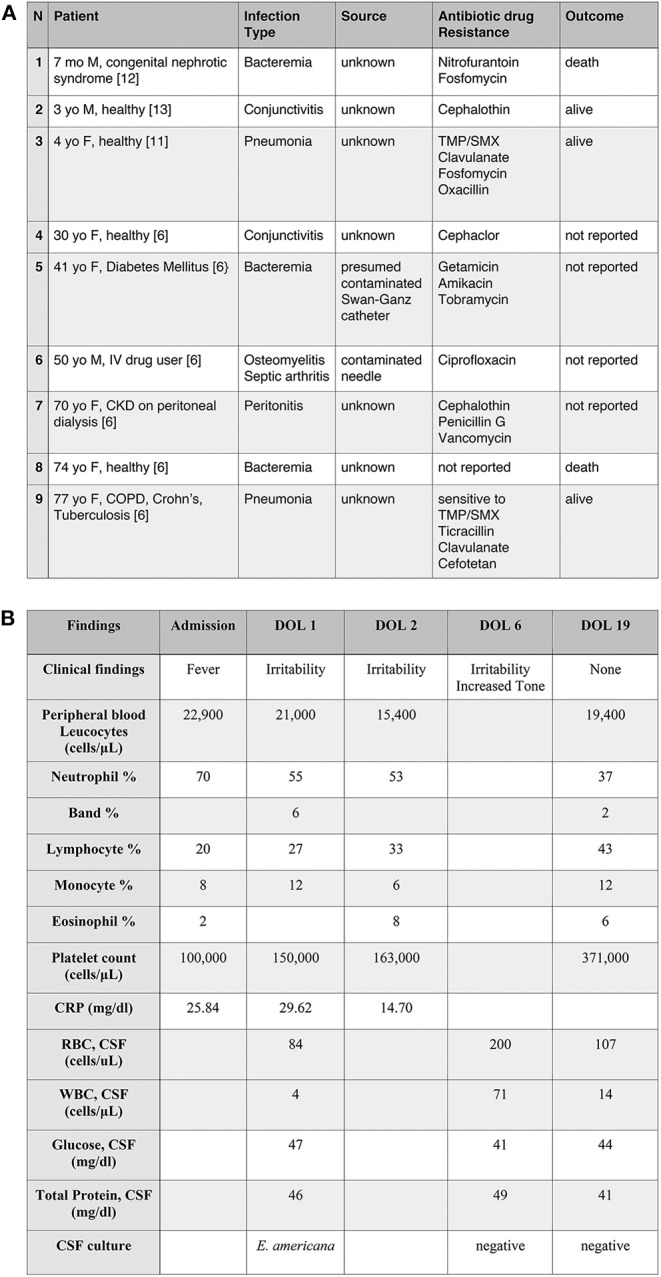
**(A)** Selected cases of *Ewingella americana* infection. Examples of clinical presentation after *E. americana* infection with the associated antibiogram. To note, the prevalence of immunocompromised patients and the presence of only three previously reported pediatric cases (case n.1, 2, and 3). TMP/SMX, Trimethoprim/sulfamethoxazole; CKD, Chronic kidney disease; COPD, Chronic obstructive pulmonary disease. **(B)** Clinical and laboratory findings in a newborn with *E. americana* meningitis.

*Ewingella americana* was first described by Grimont et al. as a new phenotypically unique cluster of 10 clinical isolates of *Enterobacteriaceae* (1). It is a rare Gram negative, member of the *Enterobacteriaceae* family, lactose fermenting, oxidase and indole negative, catalase positive and anaerobic bacteria. The bacteria grows on 5% sheep blood agar aerobically and anaerobically and on MacConkey agar with overnight incubation at 37°C (2). Most strains are methyl red positive, Voges-Proskauer positive, Simmons citrate positive, lysine decarboxylase negative, ornithine decaroxylase negative, arginine dihydrolase negative (3). *E. americana* shares the most common characteristics of *Enterobacteriaceae* but several strains do not grow well in some media recommended for the cultivation of bacteria of this family (4). The natural habitat of *E. americana* is still unknown, with some reports suggesting that the citrate solutions (prepared in the hospital for coagulations study), running water, ice baths and catheters (5) may harbor the organism. It is reported that *E. americana* can survive in water, grows well at 4C (6) and is commonly present in some vegetables (7). With regards to human samples, *Ewingella* has been found in blood, wounds, sputum, urine and feces (4). However, the detection of *E. americana* in humans as a real culprit of infection is rare. Despite rarely causing human infection, it has previously been reported as a cause of conjunctivitis, Waterhouse-Friderichsen syndrome, bacteremia, peritonitis, pneumonia and osteomyelitis, usually in immunocompromised patients and/or intravenous drug users (Figure 1A). Colonization in wound and sputum were also reported without causing clinical manifestations (4, 8). There are only three reported pediatric cases in the literature. Here, we present the first reported case of meningitis caused by *E. americana* in the youngest patient ever reported to be infected with this organism.

## Case Presentation

A newborn male was born at 40 weeks and 4/7 days gestation to a Gravida 2, Para 0, 28 years old woman. Prenatal screening tests, including group B streptococcus, Human Immunodeficiency Virus and syphilis were negative. The patient's mother was admitted for delivery at 40 weeks and 4/7 days gestation in spontaneous labor. Rupture of membranes was 27 h prior to delivery. The mother remained afebrile during the hospital stay, during and after the delivery. Infant emerged well-appearing with APGAR scores of 9 and 9 at 1 and 5 min, respectively and was placed on mother's chest for skin-to-skin contact. He was then brought to the warmer for evaluation and was noted to be febrile to 101°F. He was transferred to the neonatal intensive care unit (NICU) for further evaluation and management. Infant's temperature normalized quickly without any interventions.

As per our neonatal sepsis protocol, modified from the Kaiser Newborn Sepsis Calculator, 2015, complete blood counts (CBC) and blood culture were obtained on admission and the infant was started on ampicillin and gentamicin (9). The CBC at admission revealed WBC 22,900/uL (differential as noted in Figure 1B) and platelets of 100,000/uL.

CBC repeated a few hours later showed normal platelets of 150,000/uL, WBC 21,000/uL (Figure 1B). Initial C-reactive protein (CRP) drawn at 12 and 24 h of life were 25.84 and 29.62 mg/dl, respectively. As a result of the elevated CRPs in the context of infants' irritability, a lumbar puncture (LP) was performed with a plan to continue antibiotic therapy for seven days. At that time, anterior fontanelle was flat, reflexes and tone were normal. Vital signs, moreover, were always within normal limits without any indicator of sepsis or hemodynamics instability. Child was on full feeds per os with normal urine output.

Initial cerebrospinal fluid (CSF) profile, drawn on day of life (DOL) 1, was unremarkable with 4 WBC/uL, 84 RBC/uL, protein of 46 mg/dl and glucose of 47 mg/dl (Figure 1B). The initial blood culture was negative. CSF grew gram negative bacteria at 48 h, but the microbiology laboratory reported the results to the medical team 5 days later because it was unable to be identified. The specimen was therefore sent out to the New York State laboratory (Wadsworth Center, Albany, NY) for further speciation. While speciation was pending, a repeat LP performed on DOL 6 showed increased WBC (71/μL), RBC (200/μL), normal protein (49 mg/dl), and glucose (41 mg/dl) (Figure 1B). Antibiotics were switched to ampicillin and ceftazidime for broader Gram-negative CSF coverage. The second CSF culture was negative. The infant remained stable, afebrile, but persistently irritable. Initially, the irritability was thought to be secondary to a reflux gastroesophageal. Feeding specialist was consulted and ruled out reflux as a cause. On day of life 6, child started to present increased tone at the level of the upper and lower extremities that required evaluation and intervention by physical therapy. Tone improved prior to the infant's discharge from the NICU. The rest of the neurological examination, as well as the complete physical examination, remained unremarkable.

**Figure 2:**
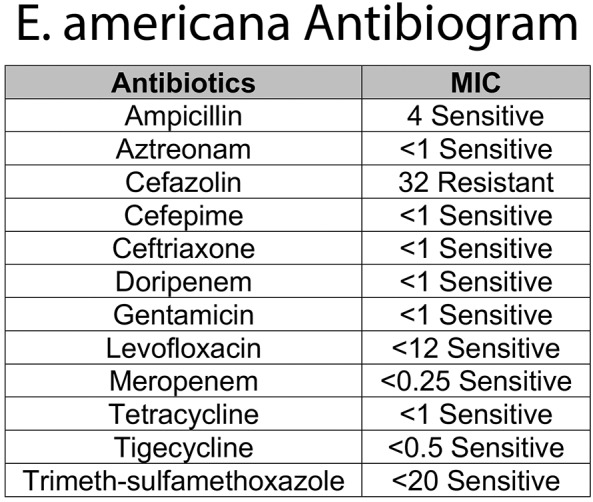
Antimicrobial susceptibility results of *E. americana* in our patient.

One week later, the final report of the initial CSF showed growth of *E. americana*, identified by MALDI-TOF bacterial identification with resistance only to cefazolin (10). Based on the sensitivity profile (Figure 2) and a better CSF penetration, the infectious disease team recommended discontinuing ampicillin and treating with ceftazidime for 21 days from the first negative CSF culture. Given the rarity of this organism, immunological workup was done and was within normal limits (IgG 656 mg/dL, IgA <15 mg/dL, IgM 16 mg/dL, CD4/CD8 ratio 1.93, Neutrophil Oxidative Burst 285, CD4 48%, CD8 24.9%, CD19 14.3%). LP done prior to discharge was within normal limits with a negative CSF culture (Figure 1B).

The infant passed initial hearing screen at birth and subsequent brainstem auditory evoked response testing done prior to discharge was within normal limits. A brain MRI performed at 2 weeks of life to exclude presence of brain abscess or subdural effusion (known complication of Gram negative meningitis) showed findings consistent with mild leptomeningitis, normal ventricles with no epidural or subdural effusion, no brain abscess or intracranial hemorrhage. The infant completed 21 days of antibiotic therapy and was discharged home. He has since followed up with neurology and infectious disease and is doing well. At 1 year follow-up, baby had normal physical examination with no other history of infections. Thereby decision was taken that a repeat immunological work up was not warranted. He had a normal electroencephalogram (EEG) and is meeting all developmental milestones at the 24 months of age follow up visit.

## Discussion

*Ewingella americana* is an emerging, Gram negative bacterium identified for the first time in the ‘80s as an opportunistic agent. Its pathogenic significance and niches of the reservoir have not been clarified yet. *E. americana* is the only species in the genus. This microorganism has rarely been found in clinical samples and clinical infections due to this Gram negative bacilli with a low pathogenic potential are rare. Cases of clinical infections in otherwise healthy patients are very rare, usually mild and limited to infections with rapid favorable outcomes (11). On the other hand, severe clinical manifestations such as bacteremia or osteomyelitis have been reported in subjects who, due to the presence of an underlying acute or chronic severe clinical condition, can be considered immunocompromised and therefore at risk of invasive infections.

Infections in children are even more infrequent than in adults and have been described in only 3 cases. The first case was a 7 months old immunocompromised male with a history of congenital nephrotic syndrome who presented with bacteremia secondary to *E. americana*. The patient ultimately became septic and passed away (12). The second and third cases were in previously healthy children who developed pneumonia (11) and conjunctivitis (13), respectively, with *E. americana* and recovered fully after antibiotic therapy. Overall, the cases previously reported in the pediatric population confirm the low virulence of the organism, leading to severe infections only in immunocompromised children.

In the presented case, *E. americana* was isolated in the CSF of a full term male infant admitted to the NICU for elevated temperature at birth that resolved on its own without any further febrile episode. Considering the rarity of the bacteria and the report to the medical team of its growth at 5 days after the LP, concern for a possible contamination from low inoculum of *Ewingella americana* was raised. However, given the onset of the growth at 48 h, the clinical picture of fever with irritability, the subsequent increased tone and MRI findings of leptomeningitis along with the development of CSF pleocytosis (in the presence of an unremarkable RBC count) in the second CSF sample, we believe that this was a true infection. The first CSF showed normal cell count, highlighting the low virulence of this organism. The second CSF showed pleocytosis in the context of unremarkable RBC count, indicating subsequent inflammatory reaction that required a few days to become evident. Finding no initial CSF abnormality with positive CSF culture was already described in the literature as a possible sign of early or developing bacterial meningitis (14). In this case, the inflammatory response detected in the second LP was further noted on the brain MRI, whose findings were consistent with leptomeningitis (15). The second CSF culture was likely negative because the child had been on effective antibiotics for the preceding 6 days.

Because severe infections caused by *E. americana* usually develop in elderly or immunocompromised patients and are rare in the younger and healthy population, a full immunological workup was done with results within normal limits for the neonatal period. This suggests that newborns may be more susceptible to severe infections caused by *E. americana*. Because of the organism's preferential growth in liquid, it is suspected that the source of infection was maternal genitourinary tract because of the maternal history of pyuria and prolonged rupture of membranes.

Finally, this is not only the first case of *E. americana* clinical infection in a newborn but also the first case of meningitis due to this organism reported in the literature. At admission, presence of fever in newborn warranted blood culture, and as the etiology could not be determined at that time, onset of antibiotic treatment with ampicillin and gentamicin. In the following 24 h of life, however, the elevated acute phase reactant serum levels with the clinical picture of fever and irritability were suspicious for meningitis so CSF culture was sent as well. Once the microbiology laboratory reported the CSF growth of an unidentifiable Gram-negative bacteria, the antibiotic therapy was changed to ampicillin and ceftazidime for a broader Gram-negative CSF coverage. When the results of antibiotic sensitivity tests became available 1 week later showing resistance only to cefazolin (Figure 2), antibiotic treatment was continued with ceftazidime alone for a total of 21 days from the first CSF culture negative. A limitation of this case is the lack of a viral panel performed in either of the CSF specimens. Shortly after this case, we started to add a PCR viral panel to be run with CSF cultures to have a better sensitivity of lumbar puncture results.

## Conclusions

This case suggests that *E. americana* infections can present in the neonatal period causing meningitis with minimal symptomatology. Increased awareness is warranted in this vulnerable population due to the emerging presence of low virulent organisms in neonatal infections and due to the emergence of resistance to first-line drugs. Finally, more reported cases and information are needed to clarify the pathogenicity of *E. americana* in the pediatric population.

## Data Availability Statement

The original contributions presented in the study are included in the article/supplementary materials, further inquiries can be directed to the corresponding author/s.

## Ethics Statement

Written informed consent was obtained from the individual(s), and minor(s)' legal guardian/next of kin, for the publication of any potentially identifiable images or data included in this article.

## Author Contributions

SM, RK, AN, and CT participated in case clinical management, data acquisition, performed the literature search, and drafted the manuscript. LK critically reviewed the manuscript for important intellectual content. All authors approved the final version of the report.

## Conflict of Interest

The authors declare that the research was conducted in the absence of any commercial or financial relationships that could be construed as a potential conflict of interest.
